# Melatonin as an Integrative Adjunct in Multimodal Analgesia: Linking Circadian Regulation, Anti-Inflammatory Modulation, and Opioid-Sparing Mechanisms

**DOI:** 10.3390/ijms27042046

**Published:** 2026-02-22

**Authors:** Nian-Cih Huang, Chih-Shung Wong

**Affiliations:** 1 Department of Anesthesiology, Tri-Service General Hospital, Taipei 114, Taiwan; niancih@hotmail.com; 2 Graduate Institute of Medical Sciences, National Defense Medical University, Taipei 114, Taiwan; 3 Department of Anesthesiology, Cathay General Hospital, #280, Section 4, Renai Road, Taipei 106, Taiwan

**Keywords:** melatonin, multimodal analgesia (MMA), chronic post-surgical pain (CPSP), circadian rhythm, opioid tolerance, Enhanced Recovery After Surgery (ERAS), neuropathic pain

## Abstract

Purpose of Review—sleep disturbance is the main complaint associated with patients who suffer acute postoperative pain. Sleep disturbance may also increase the pain sensitivity and contribute to the development and maintenance of chronic pain. The pathophysiology of pain is complex; management of perioperative pain and preventing chronic pain are challenges in clinical. Use of opioids for pain management are still a therapeutic mainstay and generally safe when taken, in a short time, for severe postoperative pain relief. For long-term use tolerance may be developed, and for their euphoric property, addiction, overdose incidents, and even death may be the social problems. Therefore, the opioid-sparing multimodal analgesia (MMA) for pain management is recommended in current postoperative pain management. The successful MMA for pain management will enhance patient recovery after surgery with less chronic CPSP and long-term opioid use disorder (OUD). The present review discusses all currently used analgesics actions and interactions, and opioid-sparing or opioid-free analgesia in perioperative pain management. Acute pain following major trauma or surgery may originate from both nociceptive and neuropathic mechanisms. Approximately 10–50% of surgical patients develop chronic postoperative pain, which not only causes persistent discomfort but also leads to functional limitations and psychological distress. Growing evidence highlights a close and bidirectional relationship between sleep and pain: pain disrupts sleep architecture, while sleep deprivation intensifies pain sensitivity and impairs recovery. This reciprocal interaction forms a vicious cycle that poses challenges for effective pain management. Melatonin—a neurohormone secreted by the pineal gland—plays a crucial role in regulating circadian rhythm and sleep–wake cycles. Beyond its chronobiotic action, melatonin exhibits anti-nociceptive, anti-inflammatory, and opioid-sparing properties. Recent preclinical studies have demonstrated that exogenous melatonin can attenuate nociceptive responses to noxious stimuli and enhance morphine analgesia while attenuating morphine tolerance. Moreover, environmental light manipulation preserving the circadian rhythm has been shown to synergistically maintain melatonin secretion, improve sleep quality, and modulate neuroimmune responses involved in pain regulation. Together, these findings suggest that circadian alignment and melatonin supplementation may represent a promising integrative approach for improving both pain control and sleep health in perioperative and chronic pain conditions. Chronic pain patients frequently experience opioid tolerance during long-term therapy, resulting in diminished analgesic efficacy and a need for escalating doses. Our recent work revealed that constant light exposure suppresses endogenous melatonin, heightens pro-inflammatory cytokines (TNF-α, IL-1β), reduces IL-10, and accelerates morphine tolerance in a neuropathic pain model. In contrast, maintaining circadian light–dark cycles or supplementing melatonin preserves melatonin rhythm, reduces glial activation, and sustains morphine antinociception. Melatonin’s co-administration not only attenuates morphine tolerance but also enhances morphine efficacy through the modulation of inflammatory and glial pathways. These findings underscore melatonin’s multifaceted role as both a chronotherapeutic and neuroprotective agent, integrating circadian regulation with pain modulation. Clinically, the application of melatonin or circadian-aligned strategies could guide personalized pain and sleep management, offering safer and more effective multimodal analgesic protocols with reduced opioid dependence and improved quality of life.

## 1. Introduction

### Literature Search Strategy and Scope

This narrative review aims to critically synthesize current evidence regarding the role of melatonin in pain modulation and its potential integration into multimodal analgesia strategies. A literature search was conducted using PubMed, Scopus, and Web of Science databases, covering studies published between January 2000 and August 2025.

Search terms included combinations of “melatonin”, “pain”, “multimodal analgesia”, “opioid tolerance”, “opioid-sparing”, “circadian rhythm”, “sleep disturbance”, and “Enhanced Recovery After Surgery (ERAS)”. References from relevant review articles were also screened to identify additional pertinent studies.

Both preclinical studies (animal and mechanistic investigations) and clinical studies (randomized controlled trials, observational studies, and meta-analyses) were included. Editorials, conference abstracts, and non-peer-reviewed articles were excluded. When discussing clinical implications, greater emphasis was placed on evidence derived from human studies, whereas preclinical findings were explicitly presented as mechanistic or hypothesis-generating observations rather than direct clinical recommendations.

## 2. Classification of Pain

For clinical and mechanistic discussion, pain is commonly categorized according to duration and underlying pathophysiology. Acute pain typically arises from tissue injury or inflammation, such as postoperative pain, whereas chronic pain persists beyond normal tissue healing and is associated with sustained neuroplastic changes within the nervous system [1].

From a mechanistic perspective, pain is broadly classified as nociceptive or neuropathic. Nociceptive pain results from activation of peripheral nociceptors by tissue-damaging stimuli, while neuropathic pain arises from injury or dysfunction of the somatosensory nervous system [2,3]. Neuropathic pain is frequently accompanied by persistent hypersensitivity, including allodynia and hyperalgesia, reflecting altered central pain processing and impaired modulatory control [4].

## 3. Progression from Acute to Chronic Pain

Chronic post-surgical pain (CPSP) represents a maladaptive transition from acute to chronic pain and is recognized as a clinically relevant postoperative complication. Its incidence varies by surgical procedure and has been reported even after relatively minor operations, such as open inguinal hernia repair, with rates of approximately 10% [5].

Accumulating evidence indicates that central sensitization plays a key mechanistic role in this transition [6]. Sustained nociceptive input can induce neuroplastic changes within the spinal cord and supraspinal structures, leading to amplification and persistence of pain signals [7,8]. At the spinal level, enhanced excitability of dorsal horn neurons—driven in part by repeated C-fiber activation and NMDA receptor-dependent mechanisms—contributes to allodynia and hyperalgesia [9]. In parallel, neuroinflammatory processes and glial activation further modify synaptic transmission, reinforcing pain chronification following tissue or nerve injury [10].

## 4. Neurochemical Mediators in Pain Processing

Pain processing is mediated by coordinated neurochemical signaling across peripheral and central nociceptive circuits. Following tissue injury, activation of peripheral nociceptors initiates afferent signaling to the spinal cord and higher central nervous system centers, where sensory input is integrated into pain perception [11,12]. At the peripheral level, excitatory neurotransmitters and inflammatory mediators released at the site of injury contribute to nociceptor sensitization and amplification of nociceptive input [13,14].

Within the spinal dorsal horn, the balance between excitatory and inhibitory neurotransmission plays a critical role in shaping nociceptive signal propagation. Excitatory mediators facilitate ascending pain transmission, whereas inhibitory neurotransmitters provide counter-regulatory control over neuronal excitability [15,16]. Persistent or excessive activation of these pathways can disrupt this balance, leading to neuroplastic changes that underlie central sensitization and chronic pain maintenance [2,6]. In parallel, inflammatory signaling cascades—including cytokine release and cyclooxygenase-dependent pathways—further modulate nociceptive processing and influence responsiveness to analgesic interventions [1,6,11].

Importantly, these neurochemical and inflammatory mechanisms constitute potential modulatory targets rather than isolated therapeutic endpoints. They provide a mechanistic framework for understanding how endogenous and exogenous factors may influence pain amplification and persistence, forming the basis for subsequent discussion of melatonin as a modulatory adjunct rather than a primary analgesic agent.

## 5. Pain Pathways

Pain arising from tissue injury is transmitted through an integrated nociceptive network encompassing peripheral afferent fibers, spinal dorsal horn circuitry, and supraspinal processing centers [12]. Nociceptive input is conveyed primarily via Aδ and C fibers and undergoes dynamic modulation within the spinal cord before being integrated with affective, cognitive, and autonomic components of pain at higher central nervous system levels [17,18,19,20].

Rather than functioning as a linear pathway, nociceptive processing is highly plastic and subject to modulation at multiple anatomical levels. Alterations in excitatory–inhibitory balance, neuroimmune signaling, and synaptic connectivity critically influence pain amplification and persistence, particularly in chronic pain states characterized by central sensitization [21]. These modulatory processes represent key targets for multimodal analgesic strategies, which aim to attenuate nociceptive transmission across multiple levels of the pain pathway rather than focusing on single molecular targets. Within this context, endogenous neuromodulatory systems and agents that influence oxidative stress, inflammation, and synaptic plasticity have attracted increasing attention. Melatonin has been proposed as one such modulatory factor, acting through antioxidant, anti-inflammatory, and central neuromodulatory mechanisms that may influence pain processing without functioning as a conventional analgesic.

## 6. Multimodal Analgesia

Effective postoperative pain control is essential for early mobilization, functional recovery, and reduction in postoperative morbidity. However, reliance on single-agent analgesic strategies is frequently limited by insufficient efficacy and dose-dependent adverse effects. Consequently, contemporary pain management emphasizes strategies that target multiple nociceptive mechanisms simultaneously.

Multimodal analgesia (MMA), a core component of Enhanced Recovery After Surgery (ERAS) protocols, involves the coordinated use of analgesic agents with distinct mechanisms of action to achieve superior pain control while minimizing adverse effects associated with high-dose monotherapy [13,22]. By engaging both peripheral and central pain pathways, MMA reduces nociceptive amplification and improves analgesic efficacy.

A principal objective of MMA is opioid sparing, particularly in the perioperative setting where opioid-related adverse effects and the risk of tolerance or dependence remain major clinical concerns. Epidemiological data highlighting the contribution of opioids to drug-related mortality underscore the importance of strategies that limit opioid exposure whenever feasible [23]. Accordingly, MMA is especially beneficial for patients with opioid tolerance or heightened vulnerability to opioid-related complications.

Rather than eliminating opioids entirely, MMA seeks to optimize analgesic balance by reducing opioid requirements through complementary pharmacological and non-pharmacological approaches, thereby improving safety and clinical outcomes [24]. This integrative framework provides a rational basis for exploring adjunctive modulatory factors that influence pain processing, inflammation, and recovery within multimodal pain management paradigms.

## 7. Enhanced Recovery After Surgery (ERAS) Protocols

Enhanced Recovery After Surgery (ERAS) protocols have been widely implemented across diverse surgical specialties with the overarching goals of optimizing perioperative care, reducing opioid exposure, and accelerating functional recovery. A central component of ERAS pathways is the integration of multimodal analgesia (MMA), which targets multiple nociceptive mechanisms to improve pain control while minimizing opioid-related adverse effects [22,25].

Across surgical disciplines—including colorectal, orthopedic, thoracic, gynecologic, urologic, and head and neck surgery—ERAS-based pain management strategies share several core principles. These include early mobilization, early resumption of oral intake, and the preferential use of opioid-sparing analgesic regimens supported by regional anesthesia techniques and non-opioid systemic agents [26,27,28]. Although specific analgesic modalities vary according to surgical context, the underlying rationale remains consistent: attenuation of nociceptive amplification, preservation of physiological function, and reduction in opioid-related complications.

Importantly, ERAS frameworks emphasize individualized, mechanism-based pain management rather than reliance on a single pharmacological approach. This paradigm provides a relevant clinical context for exploring adjunctive modulatory factors that may influence pain processing, inflammation, sleep quality, and recovery trajectories within multimodal perioperative care, while recognizing that the clinical integration of such adjuncts requires rigorous human evidence.

Within this framework, the potential positioning of melatonin as a supportive, non-analgesic adjunct across different surgical specialties is summarized in Table 1. Importantly, this table is intended to provide a conceptual overview of how circadian regulation, sleep quality, and neuroinflammatory modulation may intersect with established ERAS-based multimodal analgesic strategies. The proposed roles of melatonin are hypothesis-generating and not meant to imply routine clinical implementation, as robust human evidence supporting direct analgesic or opioid-sparing effects remains limited.

## 8. Regional and Systemic Analgesia in Multimodal Pain Management

Regional anesthesia constitutes a cornerstone of multimodal analgesia and is a key component of Enhanced Recovery After Surgery (ERAS) protocols, providing effective pain control while reducing perioperative opioid requirements and associated adverse effects [25]. Common regional techniques include neuraxial anesthesia and peripheral nerve blocks, which attenuate nociceptive input at its source and modulate central pain processing.

Clinical evidence supports the use of epidural and thoracic epidural analgesia for major abdominal and thoracic procedures, where these techniques have been associated with improved analgesia, reduced stress responses, and lower rates of respiratory and gastrointestinal complications [32,33]. Peripheral nerve block techniques, including paravertebral and transversus abdominis plane blocks, provide comparable analgesic efficacy with fewer hemodynamic side effects in selected surgical populations, contributing to enhanced postoperative recovery and opioid sparing [26,39,40].

In addition to regional techniques, systemic non-opioid analgesics play a critical role in multimodal pain management by targeting distinct nociceptive mechanisms. Acetaminophen and non-steroidal anti-inflammatory drugs (NSAIDs), including selective cyclooxygenase-2 inhibitors, reduce peripheral and central sensitization and are widely incorporated into perioperative analgesic regimens [30,41,42,43,44,45]. Adjuvant agents such as gabapentinoids and N-methyl-D-aspartate (NMDA) receptor antagonists further modulate excitatory neurotransmission and have been shown to reduce postoperative pain intensity, opioid consumption, and opioid-induced hyperalgesia in selected clinical settings [46,47,48,49].

Despite their central role in perioperative analgesia, opioids are associated with well-recognized adverse effects and the development of opioid-induced hyperalgesia and tolerance, particularly with repeated or high-dose exposure [50,51,52]. Contemporary pain management strategies therefore emphasize opioid-sparing approaches that integrate regional anesthesia, non-opioid systemic agents, and individualized dosing strategies to optimize analgesic efficacy while minimizing opioid-related harm [38,53,54].

Collectively, the integration of regional and systemic analgesic modalities reflects a mechanism-based approach to perioperative pain management. This framework provides a clinically relevant context for evaluating adjunctive modulatory factors that influence neuroinflammation, synaptic plasticity, and pain processing, which may further enhance the effectiveness and safety of multimodal analgesic strategies.

## 9. Circadian Rhythms, Sleep Deprivation, and Pain

Sleep and circadian rhythms are fundamental regulators of physiological homeostasis and cognitive function. Normal sleep architecture, comprising rapid eye movement (REM) and non-REM stages, is tightly coordinated with circadian timing systems that influence neuroendocrine, immune, and metabolic processes [55,56]. Melatonin, synthesized primarily by the pineal gland, plays a central role in synchronizing sleep–wake cycles and circadian rhythms, with secretion patterns strongly regulated by environmental light exposure [43,57].

Circadian rhythms are orchestrated by central and peripheral oscillators, with the suprachiasmatic nucleus (SCN) acting as the principal central pacemaker [58]. Light signals detected by intrinsically photosensitive retinal ganglion cells (ipRGCs) entrain the SCN, thereby modulating downstream melatonin synthesis and release [59]. Through this pathway, alterations in light exposure or circadian alignment can disrupt melatonin signaling and broader physiological regulation.

Accumulating evidence indicates a bidirectional relationship between sleep disturbance and pain. Sleep deprivation or circadian misalignment is associated with heightened pain sensitivity and impaired endogenous pain modulation, whereas persistent pain can, in turn, disrupt sleep continuity and quality [29,44]. This reciprocal interaction contributes to a self-perpetuating cycle in which sleep disruption and pain mutually exacerbate one another, increasing vulnerability to chronic pain states [30].

Beyond its role in circadian regulation, melatonin exerts pleiotropic effects relevant to pain processing, including modulation of neuroimmune signaling and central nervous system function [45]. Dysregulation of melatonin production has been implicated in a range of pathological conditions, and experimental studies suggest that altered melatonin signaling may influence nociceptive processing and pain chronification [60]. Clinical observations further indicate that sleep disorders—such as insomnia, sleep apnea, and restless legs syndrome—are associated with an increased risk of chronic pain development [61,62].

Collectively, these findings support the concept that circadian disruption and sleep deprivation represent important modulators of pain sensitivity and persistence. This mechanistic framework provides a rationale for examining circadian alignment and melatonin-related pathways as modulatory factors in pain regulation, while recognizing that translation to clinical pain management requires careful interpretation and robust human evidence.

## 10. The Role of Melatonin in Pain Regulation

**Figure 1:**
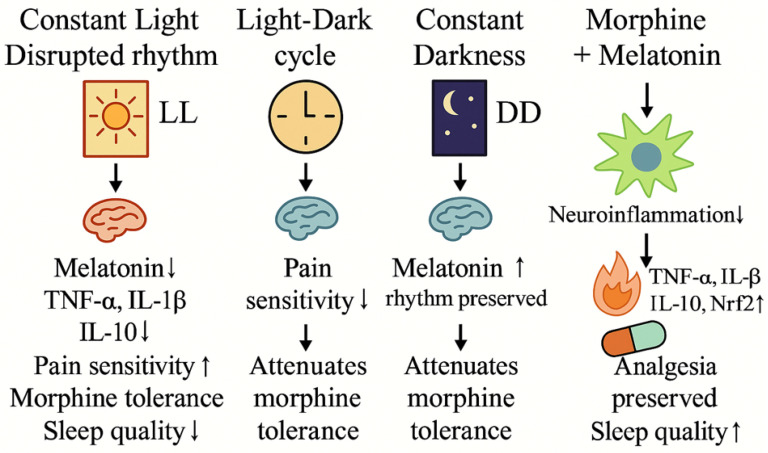
Effects of Light Conditions and Melatonin on Pain Modulation and Neuroinflammation. Constant light (LL) disrupts circadian rhythm, suppresses melatonin secretion, increases pro-inflammatory cytokines (TNF-α, IL-1β), decreases anti-inflammatory IL-10, enhances pain sensitivity and morphine tolerance, and impairs sleep quality. A normal light–dark (LD) cycle reduces pain sensitivity and attenuates morphine tolerance. Constant darkness (DD) preserves melatonin rhythmicity and attenuates morphine tolerance. Combined morphine and melatonin treatment reduces neuroinflammation, decreases TNF-α and IL-6, increases IL-10 and Nrf2 expression, preserves analgesia, and improves sleep quality. Downward arrows (↓) indicate a decrease or suppression; upward arrows (↑) indicate an increase or enhancement; vertical arrows represent directional or causal relationships between conditions and downstream biological effects.

Melatonin is best known for its role in circadian rhythm regulation; however, accumulating evidence indicates that it also functions as a pleiotropic modulator of pain-related processes beyond sleep–wake control [31]. Preclinical and mechanistic studies suggest that melatonin influences nociceptive processing through multiple pathways, including modulation of neuroinflammatory responses, oxidative stress, and central neurotransmission, as summarized in Figure 1.

Melatonin has been shown to exert anti-inflammatory effects by suppressing pro-inflammatory cytokines such as tumor necrosis factor-α (TNF-α) and interleukin-1β (IL-1β), while enhancing anti-inflammatory mediators including interleukin-10 (IL-10), thereby attenuating inflammation-associated pain signaling [63]. In addition, its potent antioxidant properties reduce oxidative stress and may limit the sensitization of peripheral and central nociceptive pathways [64]. Melatonin also interacts with several neurotransmitter systems, including serotonergic, dopaminergic, and γ-aminobutyric acid (GABA)ergic signaling, highlighting its role in central pain modulation [12].

Experimental evidence further suggests functional interactions between melatonin signaling and the endogenous opioid system. These interactions may influence opioid analgesic efficacy and tolerance development, potentially through shared pathways involving neuroinflammation and synaptic plasticity [37]. Melatonin receptors are widely expressed in both central and peripheral components of nociceptive circuits, supporting its role as a modulatory rather than primary analgesic agent [64]. Nevertheless, the translational relevance of these findings remains to be fully established, and critical questions regarding optimal dosing, timing, and clinical context require further investigation [65].

## 11. Light Manipulation, Circadian Disruption, and Pain Sensitivity

Alterations in environmental light exposure profoundly influence melatonin secretion and circadian alignment, thereby modulating pain sensitivity and neuroinflammatory responses. Experimental models demonstrate that constant light exposure disrupts circadian rhythms, suppresses endogenous melatonin production, and is associated with increased expression of pro-inflammatory cytokines, heightened pain sensitivity, accelerated opioid tolerance, and impaired sleep quality. In contrast, physiological light–dark cycling or constant darkness preserves melatonin rhythmicity and has been associated with reduced nociceptive sensitivity and attenuation of opioid tolerance in preclinical settings [34].

Furthermore, co-administration of melatonin in experimental pain models has been shown to mitigate neuroinflammatory signaling and oxidative stress, enhance endogenous antioxidative defense systems, and preserve opioid analgesic efficacy. In particular, activation of antioxidative enzymes and suppression of inflammatory pathways have been implicated in melatonin-mediated attenuation of morphine tolerance and inflammation [35], while circadian light manipulation further modulates these effects by maintaining melatonin rhythmicity and circadian alignment [34]. Collectively, these observations support a mechanistic link between circadian regulation, melatonin signaling, and pain modulation, while emphasizing that the current evidence is largely derived from preclinical studies and should be interpreted as hypothesis-generating rather than definitive clinical evidence.

## 12. Clinical Evidence of Melatonin in Pain Management

Although preclinical studies provide a biological rationale for melatonin as a modulator of nociceptive processing and opioid responsiveness, translation into clinical pain management remains limited. To date, most human studies evaluating melatonin have focused on perioperative settings or chronic pain syndromes, frequently assessing secondary outcomes such as sleep quality or anxiety rather than pain intensity or opioid consumption as primary endpoints [29,30].

## 13. Perioperative and Acute Pain

Several randomized controlled trials have examined perioperative melatonin administration, most commonly as a premedication. Oral melatonin, typically administered at doses ranging from 3 to 10 mg prior to surgery, has been associated with reduction in preoperative anxiety and improvement in postoperative sleep quality [36]. Effects on postoperative pain management and opioid consumption, however, have been inconsistent and generally modest.

Systematic reviews and meta-analyses indicate that any observed reduction in early postoperative pain scores is highly variable across surgical populations and study designs, and clinically meaningful opioid-sparing effects have not been consistently demonstrated [36]. Importantly, many perioperative studies were not primarily designed to evaluate analgesic efficacy and were limited by small sample sizes, heterogeneous surgical procedures, and variability in anesthetic and analgesic protocols. Consequently, current evidence does not support routine incorporation of melatonin as an analgesic component of ERAS pathways.

## 14. Chronic Pain Conditions

Clinical evidence in chronic pain populations is also limited. A phase II randomized controlled trial in patients with fibromyalgia reported improvement in pain scores and enhancement of descending endogenous pain inhibitory pathways following melatonin supplementation, suggesting a potential central modulatory effect [66]. However, these findings have not been consistently replicated across other chronic pain conditions.

At present, direct clinical evidence supporting the efficacy of melatonin in neuropathic pain, chronic post-surgical pain, or modulation of opioid tolerance in humans remains sparse. Most available studies report improvement in sleep quality, mood, or fatigue rather than robust or sustained analgesic effects [29,30]. Given the well-established bidirectional relationship between sleep disturbance and pain, such indirect benefits should be interpreted cautiously and not equated with primary analgesic efficacy.

## 15. Clinical Interpretation and Limitations

Collectively, available human data suggest that melatonin may confer modest, context-dependent benefits on pain-related outcomes, primarily through effects on sleep quality, circadian alignment, and anxiety rather than direct suppression of nociceptive signaling [31]. Substantial heterogeneity in dosing regimens, timing of administration, patient populations, and outcome measures currently precludes definitive clinical recommendations. Accordingly, melatonin should be regarded as a supportive, hypothesis-generating adjunct within multimodal analgesia rather than an evidence-based analgesic intervention [31]. Well-designed, adequately powered randomized controlled trials focusing on clinically meaningful pain outcomes, opioid consumption, and long-term recovery are required to clarify its potential role in clinical pain management.

To further illustrate this concept, Table 2 summarizes a conceptual framework for the potential positioning of melatonin across different perioperative phases within ERAS-based multimodal analgesia. Importantly, the roles described are hypothesis-generating and primarily informed by preclinical and mechanistic evidence, with clinical effects largely indirect and mediated through sleep regulation, circadian alignment, and modulation of inflammatory and stress-related pathways. The table is not intended to imply established analgesic efficacy or routine clinical implementation.

## 16. Clinical Implications and Conclusions

Multimodal analgesia (MMA) constitutes a cornerstone of contemporary perioperative pain management, particularly a critical role in ERAS protocols, emphasizing mechanism-based combinations of pharmacological and non-pharmacological strategies to achieve better analgesia, while minimizing opioid dosage and related adverse effects. Within this framework, melatonin has emerged as a biologically plausible modulatory adjunct rather than a primary analgesic agent.

Preclinical and translational studies suggest that melatonin may influence pain-related processes through modulation of neuroinflammatory signaling, oxidative stress pathways, and central neuromodulatory systems, including MT1/MT2 receptor-mediated mechanisms and Nrf2-related antioxidant responses [36,43,66]. Limited clinical observations further indicate that melatonin supplementation may enhance certain aspects of perioperative recovery, particularly sleep quality and anxiety, which may indirectly influence pain perception [67]. However, evidence supporting consistent opioid-sparing effects or direct analgesic efficacy in humans remains insufficient [51,52]. In clinical studies, melatonin has most commonly been administered orally at doses of 3–10 mg in perioperative or chronic pain contexts. Marked heterogeneity in study design, dosing strategies, timing of administration, and outcome measures limits the translational applicability of existing data. As such, the clinical integration of melatonin into MMA or ERAS protocols remains preliminary.

In summary, MMA provides a robust platform for opioid-sparing, patient-centered pain management. Within this context, melatonin represents a promising avenue for further investigation targeting circadian regulation, sleep–pain interactions, and neuroinflammatory modulation. Rigorous clinical trials and comparative effectiveness studies will be essential to define whether these mechanistic and supportive effects translate into clinically meaningful benefits in perioperative and chronic pain management [68,69].

## Figures and Tables

**Table 1:** ERAS Analgesic Strategies Across Surgical Specialties and Conceptual Positioning of Melatonin.

Specialty	Standard MMA/ERAS Analgesia	Pain Challenges	Potential Supportive Role of Melatonin
Colorectal	Epidural or TAP block, NSAIDs, acetaminophen [25,26]	High inflammatory burden; postoperative ileus	Modulation of inflammatory signaling and circadian rhythm that may indirectly influence pain-related recovery [29,30,31]
Orthopedic	Regional nerve blocks, gabapentinoids, NSAIDs [22,27]	Severe early postoperative pain; sleep disruption	Support of sleep quality and circadian alignment, potentially facilitating postoperative rehabilitation [29,30]
Thoracic	Thoracic epidural or paravertebral block, acetaminophen [28,32,33]	Respiratory compromise; opioid-induced hyperalgesia	Antioxidant and neuroinflammatory modulation observed in experimental models [34,35]
Gynecologic	TAP block, NSAIDs [22,24]	Visceral pain; opioid-related nausea	Anxiolytic and sleep-related effects that may improve perioperative comfort [29,31,36]
Urologic	Epidural or spinal anesthesia combined with NSAIDs [24,25]	Catheter-related discomfort	Possible interaction with opioid-related signaling suggested by preclinical studies [31,35,37]
Head and Neck	Acetaminophen and NSAIDs with limited opioid use [22,38]	Postoperative sleep disturbance	Circadian rhythm support and sleep stabilization during recovery [29,30]

**Table 2:** Conceptual integration of melatonin within multimodal analgesia (MMA) across the perioperative period.

Phase	Key MMA Components (ERAS)	Proposed Role of Melatonin	Mechanistic Rationale (Primarily Preclinical)	Potential Supportive Effects
Preoperative	Patient education, anxiolytics, gabapentinoids, acetaminophen [22,24,25]	Supports circadian alignment and reduces preoperative anxiety	Activation of MT1/MT2 receptors in SCN and limbic regions; modulation of circadian rhythm; antioxidant and anti-inflammatory signaling (e.g., Nrf2 activation, NF-κB suppression) [31,43,45]	Improved sleep readiness; reduced preoperative anxiety [29,36]
Intraoperative	Regional anesthesia, opioid minimization, NMDA antagonists, NSAIDs [22,24]	May modulate neuroinflammatory and oxidative stress responses during surgical stress	Regulation of redox balance and neuroinflammatory pathways; interaction with opioid-related signaling shown in experimental models [34,35,37]	Stabilization of perioperative physiological stress responses (hypothesis-generating) [31]
Postoperative	Opioid-sparing MMA, regional techniques, sleep hygiene [22,25]	Supports postoperative sleep quality and recovery processes	Modulation of cytokine signaling (e.g., IL-1β, TNF-α); glial activity regulation observed in preclinical studies [2,30,34,35]	Improved sleep quality; supportive recovery environment [29,30]

## Data Availability

No new data were created or analyzed in this study. Data sharing is not applicable to this article.
